# Understanding the Burden on Caregivers of People with Parkinson's: A Scoping Review of the Literature

**DOI:** 10.1155/2014/718527

**Published:** 2014-09-14

**Authors:** Rozina Bhimani

**Affiliations:** St. Catherine University, 2004 Randolph Avenue, No. 4250, St. Paul, MN 55105, USA

## Abstract

Caregivers are healthcare assets because they care for patients at home; however, when clinicians focus solely on patients, caregivers' needs may not be recognized. The purpose of this scoping literature review is to identify the burdens on caregivers of people with Parkinson's disease. CINAHL and PubMed databases were searched to locate thirteen original articles, one systematic review, and one meta-analysis within the last five years that highlighted caregivers' burdens. Results indicate the need to identify practical interventions that decrease caregivers' physical, psychological, and socioeconomic burdens. Correlates of Parkinson's caregiver burdens are not clearly available. Caregivers' contextual demographic information is missing, as is an understanding of how caregivers negotiate day-to-day caregiving activities. Gaps exist about how caregivers reconcile multiple medications and manage rehabilitation needs of the patient at home. A recommendation for practice is a systematic evaluation of the caregivers' capacity at the time of clinic visit.

## 1. Introduction

Parkinson's disease is a complex neurodegenerative progressive disease process which results in a broad spectrum of clinical manifestations such as rigidity and tremors that affect mobility; vivid dreaming and hallucinations that can affect sleep quality; and confusion and depression that affect psychological well-being [1, 2]. These symptoms can significantly limit the patient's ability to take part in activities of daily living and participate in social and recreational interests, thereby adversely affecting quality of life [3].

The insidious onset of disability often requires people with Parkinson's to require care in multiple settings. They may receive outpatient occupational, physical, speech, and recreational therapies coupled with care provided by family members and friends at home. These informal caregivers play an important role in keeping individuals with Parkinson's disease engaged in life, which ultimately can improve their quality of life. Informal caregivers of Parkinson's patients are often called “care-partners” to highlight the fact that the disease process has an impact on the caregivers as well [4].

## 2. Significance

Approximately one in six individuals worldwide are afflicted with some sort of neurological condition. An estimated seven to ten million people have Parkinson's disease worldwide. In the United States, one to three million people are afflicted with Parkinson's [5, 6], more than multiple sclerosis, amyotrophic lateral sclerosis, and muscular dystrophy combined. Most people diagnosed with Parkinson's are in their sixties; however, it can affect individuals as early as in the third or fourth decade of life. The incidence and prevalence of this neurological condition increase with age [5, 7]. At least one caregiver is required to care for a Parkinson's patient. Parkinson's disease costs United States' economy an estimated $25 billion per year in Social Security payments, medical treatments, and lost income. Medication alone can cost one patient about $2,500 per year while medical treatment can cost up to $100,000 [6], even though most Parkinson's patients receive care from informal caregivers, such as a spouse or child [8–10]. Concurrently, in the United States, informal caregivers of people with a disability (all illnesses) provide $375 billion dollars' worth of support by keeping patients at home instead of moving them into residential care [11]. This care, which is neither accounted for nor reimbursed in the healthcare economy, comes at a significant cost to the informal caregivers. For example, some caregivers may give up jobs, leisure time, and social activities to take care of their loved ones. Healthcare professionals often do not recognize the burdens of caregivers, because their focus is usually solely on the patient. Therefore, it is crucial that healthcare professionals gain a clear appreciation of the role and burdens of informal caregivers, including how the care they provide relates to healthcare costs.

## 3. Overview of Parkinson's Disease

Parkinson's is a disease of the brain that affects multiple body systems. Dopamine, a key catecholamine neurotransmitter in the substantia nigra of the middle brain and a requirement for the body's movement and coordination, is slowly depleted. Usually Parkinson's patients present with at least one of four cardinal clinical symptoms: tremor, bradykinesia (slow movements), rigidity, and postural instability. Because of Parkinson's substantial impact on the motor system, it is classified under a general umbrella term of movement disorder. It is important to understand that not all Parkinson's patients present with motor symptoms initially. Symptom experiences range from decreased physical capacity (mobility and activities of daily living), psychological or nonmotor fluctuations (depression, anxiety, apathy, and impulse control), and social changes (isolation, economic loss, and need for caregiver) [2, 12].

The most common medication used to treat Parkinson's disease is carbidopa/levodopa in multiple dosages. When Parkinson's medications are given on time, they control Parkinson's symptoms effectively, hence known as the “on” period. In order to avoid “off” periods, when medication effects wear off, it is crucial that Parkinson's medications are administered on time. Good clinical practice requires that clinicians are very cognizant of their patient's medication time schedule and strictly adhere to it during any healthcare organizational stay. However, to ensure strict adherence to the medication schedule, informal caregivers at home must also ensure the correct timing of medications, in order to avoid unpleasant symptoms for patients. Informal caregivers must be attuned to changes in management of symptoms and disease to avoid hospitalization and residential care [13, 14]. Furthermore, informal caregivers must be persistent and vigilant with the patient's rehabilitation needs at home. This consistent attentive care of people with Parkinson's may place multiple demands on caregivers.

## 4. Scoping Review Question

Scoping reviews use a wide range of evidences with a broad review question to provide answers, focusing on evidence that can easily be translated into practice. This review follows the definition of a scoping review for mixed methodology established by Gough et al. [15] and Levac et al. [16]. According to these authors, the scoping review is a precursor to a systematic review, undertaken in disciplines such as rehabilitation science where the availability of randomized control trials is scarce. This type of review maps and reinterprets evidence analytically. The scoping research question that guided this inquiry was “What are the burdens of informal caregiving in Parkinson's disease?”

## 5. Database Search

A scoping review of the literature from 2008 to 2013 was conducted on the burdens of informal caregivers for people with Parkinson's disease. Original research articles, meta-analysis, and systematic reviews from CINAHL and PubMed databases were included. The keywords used were “caregiver,” “Parkinson,” “neurological disease,” and “burden.” Inclusion criteria consisted of literature in English, published within the past five years, which focused on the burdens of caregivers. Studies were included if they focused on the burdens experienced by caregivers for other neurological disease processes, such as Alzheimer's disease, as long as they also included Parkinson's disease. Exclusion criteria consisted of studies that focused on Parkinson's patient issues, disease progression, treatments, and interventions. Thirteen research articles met these criteria and were included in this review (see Tables 1 and 2). Additional evidence from systematic review and meta-analysis was included to gain the overall understanding of the state of the science on this topic.

## 6. Scoping Review

### 6.1. Physical Domain

Parkinson's disease affects movement and thereby impairs daily functioning such as personal hygiene, ambulation, and daily routines. Impairments in movement and coordination are probable, necessitating caregiver's assistance. Informal caregivers are likely to integrate the rehabilitation needs of patients during the daily care routines.

#### 6.1.1. Activities of Daily Living

The literature is not robust in how caregivers provide care or rehabilitation at home. There is some indication that caregivers increasingly get stressed as the Parkinson's disease progresses and the patients become unable to engage in activities of daily living (ADLs) for themselves [11, 17–19]. Caregivers report feeling overwhelmed by the physical demands of caregiving and often find that they are unprepared to care for the patient at home. Adaptation to new daily routines for people with Parkinson's requires disruption of the caregiver's previous routines [18, 20, 21].

#### 6.1.2. Mobility

Gait patterns for people with Parkinson's may not be stable; therefore, routine mobility activities such as getting in and out of a chair or bed and crossing the road become safety issues for caregivers. People with Parkinson's experience fear of falling and freezing while ambulating is a concern raised by the caregivers. Lack of transportation for therapies is an issue in providing appropriate and safe caregiving. The literature is silent about whether informal caregivers use daily mobility as a therapeutic opportunity for rehabilitation at home [18, 20–22].

Several methodological concerns remain. Quantitative studies are lacking power analysis, sample sizes are small, and a clear demographic picture of caregivers is not available. Most studies have identified the caregivers of many people with Parkinson's to be an elderly female spouse or middle-aged child. Although the literature identifies the age, gender, and relationships of caregivers with care recipients in some studies [11, 19], other contextual factors such as ethnicity and socioeconomic and education levels that have an impact on care are often missing. Although studies have focused on caregiver issues, inappropriate scales/measurements that evaluate Parkinson's disease process rather than the strain on caregivers raises questions of design validity. The use of unmatched case controls in Tokunaga et al. [19] is troublesome as they do not evaluate equivalent caregiving experiences.

### 6.2. Psychological Domain

Emotional and psychological stresses may be present in caregiving activities. A clear understanding of the nature, severity, and extent of psychological stressors for caregivers of people with Parkinson's requires evaluation. Parkinson's disease is classified as a motor disorder; therefore, the burden of providing physical care would be quite high. However, and surprisingly, most of the literature identifies psychological issues as being the most bothersome [1, 23]. However, Lau and Au [8] found that patients' physical dependency correlated more significantly (*r* = 0.43, *P* < 0.05) with caregiver distress than did psychological behaviors (*r* = 0.33, *P* < 0.05).

#### 6.2.1. Anxiety/Depression

Anxiety and depression are commonly present in caregivers of people with Parkinson's. Mood disturbances do occur as the strain on caregivers increases. These findings not only are statistically significant but also have significant clinical relevance in caring for the caregivers [23–25]. Furthermore, sleep disturbances leading to anxiety and depression have been reported. Interestingly, Rongve, Boeve, and Aarsland report that people with Parkinson's experience more sleep-related problems (vivid dreams) compared to Alzheimer's patients, which consequently influences caregivers' sleep patterns as well. A'Campo et al. delivered formalized education interventions concurrently for both people with Parkinson's and their caregivers to decrease the psychological burden. Findings are encouraging as caregivers were less stressed (*P* < 0.05) but depressive symptoms did not change significantly.

#### 6.2.2. Cognition and Impulse Control

People with Parkinson's often exhibit impulsive behaviors, apathy, and/or a decline in cognition, which adds to the caregiver burden. Findings indicate that psychological distress was greater in caregivers who had to manage impulse control and apathy behaviors [9]. Caregivers raised concerns about clinicians relying solely on patients' accounts of their illness experiences, noting that patients often are cognitively impaired and cannot provide reliable information [18].

A plethora of scales and surveys has studied correlates of caregiver burdens due to psychological distress in caring for people with Parkinson's disease. In the literature, there is an assumption that the severity of Parkinson's disease correlates with informal caregivers' burdens, which may not be correct. The following scales have been used to evaluate depression and anxiety in both care recipients and caregivers: Hoehn and Yar (HY) to determine Parkinson's stage, unified Parkinson's disease rating scale (UPDRS), mini-mental state exam (MMSE), geriatric depression scale (GDS), caregiver burden inventory (CBI), mood visual analog scale (MVAS), self-rating depression scale (SDS), and Parkinson's disease questionnaire 39 items (PDQ-39). Most of the studies did not comment on the reliability/validity of the instruments used [9, 23, 24] or how they have maintained rigor in qualitative studies [18, 21, 22].

### 6.3. Socioeconomic Domain

People with Parkinson's who live at home need social interaction, which means that caregivers must spend time with them. Caregivers need financial stability and the means to care for the patient. Isolation and emotional and financial strains were the outcomes for many caregivers [20, 22].

#### 6.3.1. Isolation

Caregivers voiced pronounced isolation and grieved for their previous vibrant active lifestyles. Lack of time for self-care and continued focus on patients depleted their energy [20, 22]. Some caregivers did not use palliative care for respite because they felt emotional distress at having to acknowledge the death and dying process. Informal caregivers often considered palliative care services to be synonymous with hospice care, so they did not avail themselves of this service option [21]. Change in relationship dynamics was a source of discomfort, especially in spousal relationships where the caregiver was used to getting reciprocal attention and affection from their spouse. This factor was mitigated if caregivers felt attention was mutual in the relationship. Negative mutuality was associated with lower functional capabilities of the care recipient, less caregiving experience, and depressive symptoms for caregivers [10].

#### 6.3.2. Financial Strain

Caregivers of people with Parkinson's experience significant financial concerns. They were frequently unaware of available resources to lessen their economic burden. Caregivers were often women who quit their jobs to care for their spouse or retirees who were unable to continue part-time employment to supplement their income. Some had waited until they retired to travel, but financial constraints limited their ability to pursue leisure activities. Adult children of care recipients often had to miss work to care for their parents [18, 20, 21]. Effective caregiving requires a constant source of health, energy, and life balance; however, caregivers tend to spend money on care recipients' needs first, leaving little or no resources to seek respite care for themselves. These studies highlight important aspects of caregiving, which are often hidden from consideration during the “care partner” journey. Most of the research was conducted outside the United States (8 European countries, 2 United States, 2 Asia, and 1 South Africa); therefore, careful consideration of the local economic context is needed to interpret these findings.

## 7. Other Evidence

Other sources of evidence included a meta-analysis that focused on the correlates of caregiver distress [8] and a systematic review that provided insights into the psychosocial intervention for caregivers of people with Parkinson's [26].

It is important to understand which caregiving aspect causes most distress to informal caregivers of people with Parkinson's. Findings from the meta-analysis [8] indicate that physiological motor symptoms and dependence in ADLs have moderate correlation with caregiver distress. Declining cognitive function and higher level of the patient's depression level have small to moderate relationship to caregiver strain. It is the caregiving intensity (hours spent on caregiving activities) which is strongly associated with the caregiver burden. Heterogeneity of studies and the lack of a robust database to search studies are concerns in this meta-analysis. However, a clear research question, appropriate and strong inclusion criteria, the use of appropriate included studies, and utilization of appropriate statistical measures provide validity to its findings.

Effective psychosocial interventions to decrease the burden of caregivers for people with Parkinson's are essential. The systematic review by Hempel et al. [26] evaluated thirty studies (24 full studies and 6 studies published as abstracts) to obtain understanding as to which psychosocial interventions would be most beneficial for caregivers. Interventions range between day care, night-sitting services, community care assessment, web-based instructional videos on caregiving tips/strategies, formal education classes, and support groups. Findings were inconsistent due to a diverse range of interventions. This appraisal is of limited value because the reviewed studies included small sample sizes and concerns exist about the rigor of study designs and scales. The review included an appropriate population with a clear research question, but the applicability of findings in clinical practice is compromised due to inconsistent results.

## 8. Discussion

Analysis of the existing evidence does identify gaps in the literature. Information about race/ethnicity and socioeconomic and education levels are important considerations in the evaluation of the caregiver burden. Most caregivers are elderly females who are not equipped for caregiving activities due to physical or psychosocial constraints. Male caregivers are conspicuously lacking from the literature demographics. Most studies were of a correlational design; therefore, the cause and effect of Parkinson's disease and caregivers' burdens cannot be established with confidence. Scales and instruments are heavily utilized in most quantitative studies where an overabundance of data confuses the findings and interpretations. This becomes more concerning when scales are used to evaluate Parkinson's disease progression, physical functioning, and psychological symptoms as correlates for the caregiver burden without understanding other background information such as the functional capacity of caregivers themselves.

Gaps exist in the literature about what motivates caregivers to assume that role and how they negotiate day-to-day stressors. Informal caregivers are primarily responsible for medication management and providing daily rehabilitation activities at home. No information is available in the literature as to how caregivers reconcile multiple therapeutic rehabilitation and medications recommendations at home that may be prescribed by different healthcare practitioners, especially if medication regimens conflict. This is an important patient safety issue that can have a profound implication on the quality of life for people with Parkinson's.

Findings are inconsistent as to whether the physical or psychosocial domains of caregiving are more burdensome. Clear evidence about interventions that can decrease the caregiver burden is not available. Caregivers do suffer from isolation and may incur caregiving costs that may not be tangible. The literature hints at financial strains and burdens on caregivers; however, quantification is not available.

## 9. Implication for Practice

Parkinson's disease falls under the umbrella of a movement disorder; therefore, the focus of available caregiving resources is usually on the physical aspects of care. Clinicians must take a holistic view in order to understand the burden of providing informal care to people with Parkinson's disease. They need to understand that informal caregivers must respond to care-recipients' needs in all aspects of life. Because not all Parkinson's patients suffer from all symptoms, resources for caregivers must match their needs. Therefore, a systematic evaluation of caregivers' capacity at the time of clinic visit is recommended to ensure that caregivers are properly supported in their endeavors. It is crucial that caregivers are educated not only about the disease processes but also about how to manage symptoms and experiences and locate financial resources that may decrease the caregiving burden. Inclusion and availability of psychologists in an interdisciplinary team in a clinical setting are essential for both caregivers and care recipients to manage anxiety, depression, and stress.

## 10. Future Recommendations

Many unanswered questions require further research. Clear understanding of the caregiver burden and how this role is negotiated in daily life requires careful evaluation. Robust study design and appropriate sample size that account for detailed demographic information are essential to a caregiver profile. Medications and rehabilitation management patterns by informal caregivers at home require further investigation. Intervention research that focuses on decreasing caregivers' physical, psychological, social, and financial burdens is needed to gain clarity. Concurrent evaluation of the needs of people with Parkinson's and the caregiver's capacity to provide care at the time of the clinical exam is important to support caregiving activities. The available evidence does not provide any concrete or practical information to lessen the caregiver burden. The state of the science is evolving in this area; therefore, a change in practice based on sound research evidence requires further research in this area. An intentional focus on understanding the burdens on informal caregivers of people with Parkinson's disease is needed to improve their quality of life.

## 11. Conclusion

The caregiving burden may vary in different neurological diagnoses; therefore, individual caregivers may need different interventions and strategies that meet their needs in caring for loved ones. Social justice demands that human dignity must be maintained and care must be provided to individuals who are vulnerable. Ethical considerations call for advocacy and compassion for patients, families, and caregivers. Caregivers are healthcare resources, and, therefore, it is essential that caregivers must be included in healthcare transactions and their contributions in care partnering must be made transparent.

## Figures and Tables

**Table 1:** Quantitative studies.

Study (author, year)	Purpose	Population/ sample	Research design	Intervention	Comparison	Outcome measures/ scales	Results
A'Campo et al. (2010) [24]	Formative evaluation of standardized psychosocial education program on quality of life	Caregivers *n* = 137 Parkinson's patients *N* = 151	Quasiexperimental design	8 weeks parallel program for Parkinson's patient and caregivers	None	MMSE, BELA-P-k, BELA-A-k, Bb, PDQ-39, SDS, Nfh, EQ-5D VAS, and Mood VAS	Mood, social, and emotional functioning and achievement capabilities improved significantly (*P* < 0.05). Depression did not improve

Carter et al. (2008) [1]	Understand motor and nonmotor symptom impact on caregiver strain	219 spouses, mean age: 66.7	Correlational design	None	None	FCI, CES-D, and UPDRS	Nonmotor symptoms cause ×2–4-fold increase in burden (*P* < 0.05)

D'Amelio et al. (2009) [23]	Determine predictors of caregiver burden	40 Parkinson patients and caregivers	Correlational design	None	None	CBI, HY, GDS, NPI, UPDRS-ME, and MMSE	Mental symptoms (*P* = 0.03) and Parkinson's severity of disease (<0.0001) correlated with caregiver distress

Kelly et al. (2012) [11]	Determine HRQoL in people with Parkinson's and its effect on caregiver strain	97 caregiver dyads 84% spouse (part of larger RCT study)	Cross-sectional correlational design	None	None	EQ-5D, PDQ-39, MCSI, 6MWT, and HY	Good HRQoL of PD patients correlated with low caregiver strain (rho 0.43, *P* < 0.001)

Leroi et al. (2012) [9]	Determine care burden in apathy and impulse control in Parkinson's	71 carer dyads	Cross-sectional correlational design	None	Control group	UPDRS, HY, BIS-11, AES-C, ZBI, LEDD, and HADS	Care burden is significant in impulse control (*P* = 0.002 and *P* = 0.004)

de Villiers et al. (2008) [17]	Investigate needs, roles, and experiences of primary caregivers in Parkinson's	126 participants 77% female 27% male	Descriptive quantitative	None	None	Developed scale: no name	Isolation (57%) Lack of time (47%) Felt powerless (45%) Felt stress (43%) Finance issues (40%) Physically drained (32%)

Rongve et al. (2010) [25]	Identify sleep disturbances in subtypes of dementia and explore clinical correlates	151 participants	Cross-sectional comparative design	None	Alzheimer's disease	NPI, Epsworth Sleepiness Scale, MSQ, MADRS, REM,	More sleep disturbances in PD (89%) versus Alzheimer's (64%). *P* = 0.008
Shim et al. (2011) [10]	Understand correlates of care mutuality in Parkinson's and Alzheimer's disease	152 dyads for Parkinson's and Alzheimer's 91 control (16% attrition in control)	Retrospective multilevel design	None	Alzheimer's disease and control	MSFCI, Lawton, IADL, and CESD	Longer caregiving years (*P* < 0.05), and increased IADL (*P* < 0.05) increase care mutuality; increased depression in carer decreased care-mutuality (*P* < 0.05)

Tokunaga et al. (2009) [19]	Investigate caregiver burden	54 pairs Parkinson's 48 pairs control age 65 and older	Unmatched case control design	None	Frail elderly	J-ZBI, CES-D, and DBD	Parkinson's caregiver spent less time caregiving for ADLs (2.78 hours) compared to frail elderly (11.2 hours) *P* < 0.01

**Table 2:** Qualitative studies.

Study (author, year)	Purpose	Population/ sample	Research design	Analysis method	Comparison	Rigor	Themes
Hounsgaard et al. (2011) [18]	Women's experiences of care decision and self-management in Parkinson's	10 Parkinson's caregivers	Phenomenological hermeneutic approach	Ricoeur's framework	None	Participant check-back	Learning to live as a partner; contact with health service; between power and powerlessness; change in self-management

McCabe et al. (2008) [20]	Change in work and recreational changes among people with neurological illness and their caregivers	31 Parkinson's caregivers	Interviews specific design is not mentioned	Content analysis	28 multiple sclerosis, 27 motor neuron disease, 24 Huntington's	Audit trail maintained	Changes in patient and carer work situation; feelings about changes in patient and carer work situation; impact of work changes on patients and carer social life

McLaughlin et al. (2011) [21]	Caregiver's perception of living and coping with Parkinson's	26 Parkinson's caregivers	Exploratory approach: audiotaped interviews	Miles and Huberman framework	None	Not provided	Diagnosis, information needed, coordinated and continued medical care, meaning and timing of palliative care, burdens related to caregiving, and economic implication of caring

Tan et al. (2012) [22]	Understand perceptions of Singaporean caregivers in caring for Parkinson's patients	17 Parkinson's caregivers	Part of a large mixed method sequential explanatory design	Ritchie and Spencer's framework	None	Not provided	Four themes of coping and adaptation, challenges of caregiving, effects of caregiving on the caregivers, and need for better caregiver support are reported

## References

[1] Carter JH, Stewart BJ, Lyons KS, Archbold PG (2008). Do motor and nonmotor symptoms in PD patients predict caregiver strain and depression?. *Movement Disorders*.

[2] Pretzer-Aboff I, Galik E, Resnick B (2009). Parkinson's disease: barriers and facilitators to optimizing function. *Rehabilitation Nursing*.

[3] Martinez-Martin P, Rodriguez-Blazquez C, Kurtis MM, Chaudhuri KR (2011). The impact of non-motor symptoms on health-related quality of life of patients with Parkinson's disease. *Movement Disorders*.

[4] Northwest Parkinson’s Wellness Center http://www.nwpf.org/wellness/CarePartners/default.aspx.

[5] National Parkinson Foundation http://www.parkinson.org/parkinson-s-disease.aspx.

[6] Parkinson’s Disease Foundation Statistics on Parkinson’s. http://www.pdf.org/en/parkinson_statistics.

[7] United Nations http://www.un.org/apps/news/story.asp?newsid=21689&cr=neurological.

[8] Lau K-M, Au A (2011). Correlates of informal caregiver distress in Parkinson's disease: a meta-analysis. *Clinical Gerontologist*.

[9] Leroi I, Harbishettar V, Andrews M, McDonald K, Byrne EJ, Burns A (2012). Carer burden in apathy and impulse control disorders in Parkinson's disease. *International Journal of Geriatric Psychiatry*.

[10] Shim B, Landerman LR, Davis LL (2011). Correlates of care relationship mutuality among carers of people with Alzheimer's and Parkinson's disease. *Journal of Advanced Nursing*.

[11] Kelly DH, McGinley JL, Huxham FE (2012). Health-related quality of life and strain in caregivers of Australians with Parkinson’s disease: an observational study. *BMC Neurology*.

[12] Stern MB, Lang A, Poewe W (2012). Toward a redefinition of Parkinson's disease. *Movement Disorders*.

[13] Farris S, Vernon G, Carter A, Heath S (2011). Parkinson’s disease: pharmacological strategies, a roundtable discussion. *Parkinson’s Disease Counseling Points*.

[14] Vernon GM (2009). Parkinson’s disease and the nurse practitioner: diagnostic and management challenges. *Journal for Nurse Practitioners*.

[15] Levac D, Colquhoun H, O'Brien KK (2010). Scoping studies: advancing the methodology. *Implementation Science*.

[16] Gough D, Thomas J, Oliver S (2012). Clarifying differences between review designs and methods. *Systematic Reviews*.

[17] de Villiers D, Heerden S, Nel M (2008). Roles, experiences and needs of caregivers of people with Parkinsons disease in South Africa. *South African Journal of Occupational Therapy*.

[18] Hounsgaard L, Pederson B, Wagner L (2011). The daily living for informal caregivers with a partner with Parkinson’s disease: an interview study of women’s experiences of care decisions and self-management. *Journal of Nursing and Healthcare Chronic Illness*.

[19] Tokunaga S, Washio M, Miyabayashi I, Fortin E, Shin Y-S, Arai Y (2009). Burden among caregivers of Parkinson's disease patients. *International Medical Journal*.

[20] McCabe MP, Roberts C, Firth L (2008). Work and recreational changes among people with neurological illness and their caregivers. *Disability and Rehabilitation*.

[21] McLaughlin D, Hasson F, Kernohan WG (2011). Living and coping with Parkinson's disease: perceptions of informal carers. *Palliative Medicine*.

[22] Tan SB, Williams AF, Morris ME (2012). Experiences of caregivers of people with Parkinson's disease in Singapore: a qualitative analysis. *Journal of Clinical Nursing*.

[23] D'Amelio M, Terruso V, Palmeri B (2009). Predictors of caregiver burden in partners of patients with Parkinson's disease. *Neurological Sciences*.

[24] A'Campo LEI, Spliethoff-Kamminga NGA, MacHt M, Roos RAC (2010). Caregiver education in Parkinson's disease: formative evaluation of a standardized program in seven European countries. *Quality of Life Research*.

[25] Rongve A, Boeve BF, Aarsland D (2010). Frequency and correlates of caregiver-reported sleep disturbances in a sample of persons with early dementia. *Journal of the American Geriatrics Society*.

[26] Hempel S, Norman G, Golder S, Aguiar-Ibanez R, Eastwood A (2008). Psychosocial interventions for non-professional carers of people with Parkinson's disease: a systematic scoping review. *Journal of Advanced Nursing*.

